# A Low Molecular Weight Heparin Inhibits Experimental Metastasis in Mice Independently of the Endothelial Glycocalyx

**DOI:** 10.1371/journal.pone.0011200

**Published:** 2010-06-21

**Authors:** Geerte L. Van Sluis, Max Nieuwdorp, Pieter W. Kamphuisen, Johan van der Vlag, Cornelis J. F. Van Noorden, C. Arnold Spek

**Affiliations:** 1 Department of Vascular Medicine, Academic Medical Center, Amsterdam, The Netherlands; 2 Center for Experimental and Molecular Medicine, Academic Medical Center, Amsterdam, The Netherlands; 3 Department of Cell Biology and Histology, Academic Medical Center, Amsterdam, The Netherlands; 4 Department Clinical Oncology, Academic Medical Center, Amsterdam, The Netherlands; 5 Nephrology Research Laboratory, Department of Nephrology, Nijmegen Centre for Molecular Life Sciences, Radboud University Nijmegen Medical Centre, Nijmegen, The Netherlands; Institut Pasteur, France

## Abstract

**Background:**

Some low molecular weight heparins (LMWHs) prolong survival of cancer patients and inhibit experimental metastasis. The underlying mechanisms are still not clear but it has been suggested that LMWHs (at least in part) limit metastasis by preventing cancer cell-induced destruction of the endothelial glycocalyx.

**Methodology/Principal Findings:**

To prove or refute this hypothesis, we determined the net effects of the endothelial glycocalyx in cancer cell extravasation and we assessed the anti-metastatic effect of a clinically used LMWH in the presence and absence of an intact endothelial glycocalyx. We show that both exogenous enzymatic degradation as well as endogenous genetic modification of the endothelial glycocalyx decreased pulmonary tumor formation in a murine experimental metastasis model. Moreover, LMWH administration significantly reduced the number of pulmonary tumor foci and thus experimental metastasis both in the presence or absence of an intact endothelial glycocalyx.

**Conclusions:**

In summary, this paper shows that the net effect of the endothelial glycocalyx enhances experimental metastasis and that a LMWH does not limit experimental metastasis by a process involving the endothelial glycocalyx.

## Introduction

In experimental animal models and clinical studies it has been well established that some low molecular weight heparins (LMWH) inhibit experimental metastasis and prolong survival [1], [2]. Although the underlying mechanisms are only partially understood, it has been suggested that the endothelial glycocalyx may play an important role in the life prolonging effects of LMWH in patients.

The endothelial glycocalyx is a negatively charged, organized network of membranous glycoproteins, proteoglycans and glycosaminoglycans that affects several biological processes with potential importance for cancer cell extravasation. First, the endothelial glycocalyx is essential for vascular barrier function. Its disruption by pro-inflammatory cytokines, including tumor necrosis factor (TNF-α) and glycocalyx-degrading enzymes such as heparanase and hyaluronidase, leads to increased vascular permeability [3]–[5]. Second, the glycocalyx has anticoagulant properties and thrombin generation is reduced by the glycocalyx because it stores various natural anticoagulant factors such as antithrombin, protein C and tissue factor pathway inhibitor [6]. Consequently, disruption of the endothelial glycocalyx instantly results in thrombin generation and platelet adhesion [7]. Third, through its diversity in biochemical make-up, the endothelial glycocalyx both prevents and facilitates cell adhesion to the endothelium. The size of the glycocalyx (predominantly its heparan sulphate proteoglycan and hyaluronate composition) exceeds the size of the adhesion molecules (syndecan-1, L- and P-selectin), thereby masking these proteins and preventing adhesion of among others leukocytes [8]. On the other hand, when glycocalyx bound components such as hyaluronic acid are released they may serve as ligands for the CD44 receptor expressed on many cells (including cancer cells). The glycocalyx thus plays an important role in cell adhesion to the vessel wall [9], [10]. Fourth, the glycocalyx binds growth factors and extracellular matrix components via its proteoglycan syndecan-1. Moreover, syndecan-1 modulates fibroblast growth factor-2 (FGF-2) and vascular endothelial growth factor (VEGF) activity [11]. The glycocalyx is a sink of growth factors that in general are anti-apoptotic and of VEGF that can increase endothelial permeability [12]. Overall, the endothelial glycocalyx may thus be an important player in several biological processes with potential relevance for cancer cell metastasis. The relative importance of the particular pro- and anti-metastatic effects of the endothelial glycocalyx *in vivo* remains to be elucidated however.

Interestingly, cancer cells produce enzymes that are known to degrade the endothelial glycocalyx, such as heparanase and hyaluronidase [12]–[16]. These enzymes consequently influence vascular endothelial barrier integrity, adhesive properties of the endothelial lining, cytokine production and can liberate heparan sulfate-bound growth factors thereby inducing cancer cell extravasation. As heparin, LMWHs and heparin derivatives can abolish the activity or binding of heparanase [17], [18] and hyaluronidase [19] by competing with heparan sulphates and hyaluronan [20]–[22], it has been hypothesized that LMWHs (at least in part) limit cancer progression by restoring cancer cell-induced glycocalyx damage thereby limiting cancer cell extravasation [23].

In the current manuscript, we aimed to assess whether the effect of a LMWH on experimental metastasis depends on restoration of the endothelial glycocalyx. To this end, we first determined the net effect of the endothelial glycocalyx in experimental metastasis. Next, we assessed the effect of a LMWH in the presence or absence of an intact endothelial glycocalyx to determine the contribution of the glycocalyx to the effect of this LMWH on the reduction of experimental metastasis.

## Results and Discussion

**Figure 1:**
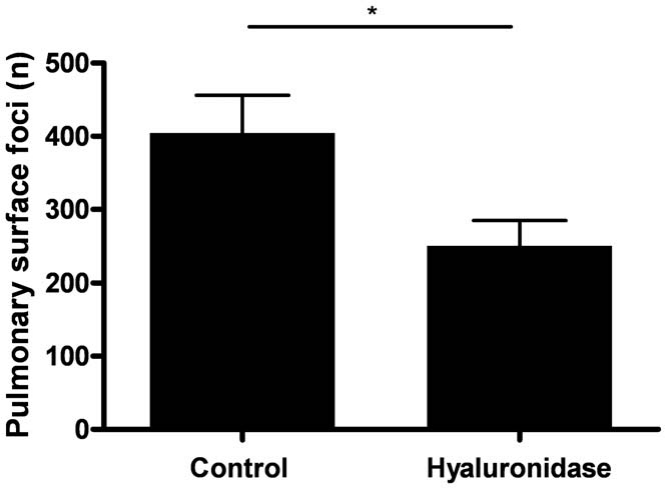
Effect of hyaluronidase on the number of B16F10 pulmonary tumor foci. C57Bl/6 mice were treated intravenously with 100U hyaluronidase 1h prior to the administration of 3.5×10^5^ B16F10 melanoma cells into the lateral tail vein. Mice were sacrificed 14 days after cancer cell injection and the number of tumor foci at the surface of the lungs was determined. Error bars represent means ± SEM (n = 8); *, p<0.05.

To assess the net effect of the endothelial glycocalyx on experimental metastasis, wild type mice were treated with hyaluronidase in order to remove hyaluronan and, in part, heparan sulphates from the endothelial glycocalyx. As it has previously been shown that one hour after hyaluronidase treatment vascular leakage is evident [4], B16F10 melanoma cells were injected intravenously 1h after intravenous hyaluronidase or saline administration. Experimental metastases in the lung were examined 14 days later. As shown in Figure 1, the number of pulmonary tumor foci was significantly reduced by approximately 30% after hyaluronidase treatment as compared to the saline injected control group. Enzymatic degradation of the glycocalyx (at least of its hyaluronan component) thus limits experimental metastasis suggesting that the net effect of the glycocalyx is pro-metastatic. These data imply that hyaluronidase-induced endothelial barrier disruption and consequent increased vascular permeability that would promote cancer cell extravasation is counteracted by the loss of specific adhesion molecules and/or growth factors from the glycocalyx. However, it should be realized that hyaluronidase treatment may not only destroy the endothelial glycocalyx but may also trigger the immune system which would reduce the number of cancer cells in the circulation [24], [25]. Furthermore, hyaluronidase increases circulating levels of hyaluronan oligomers which are known to limit cancer progression [10]. In addition, one could argue that systemic hyaluronidase treatment may also target the glycocalyx of cancer cells and this might be particularly relevant because impairment of the glycocalyx makes the cancer cell vulnerable to the immune system [26]. However, circulating hyaluronidase levels at the moment of cancer cell inoculation are rather low due to the short half-life of hyaluronidase (i.e. 2.7 minutes in rat plasma [27], resulting in a circulating level of below 0.0001 U) suggesting that the observed effect is not dependent on destruction of the cancer cell glycocalyx.

**Figure 2:**
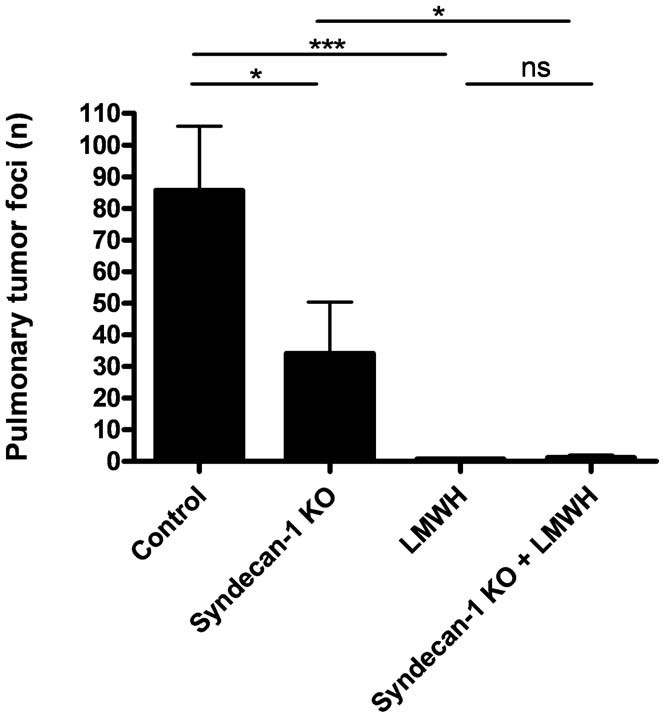
Pulmonary tumor foci formation in syndencan-1 −/− versus wild type mice with and without treatment with LMWH. Syndecan-1 −/− and wild type mice were administered 2.0×10^5^ B16F10 melanoma cells into the lateral tail vein. One group of mice was treated with LMWH (15 mg/kg enoxaparin ) prior to the administration of B16F10 melanoma cells and LMWH treatment was repeated after 6, 12 and 24 h. Mice were sacrificed 14 days after cancer cell injection and the number of tumor foci at the surface of the lungs was determined. Error bars represent medians ± interquartile range (n = 8), * p<0.05; *** p<0.001.

To confirm the pro-metastatic effect of the glycocalyx and to exclude “side effects” like acute immunological responses [24], [25] and/or increased hyaluronan oligomers of hyaluronidase treatment that may also be responsible for the observed reduction in cancer cell extravasation, we assessed the effect of a genetically impaired glycocalyx on cancer cell extravasation. To this end, syndecan-1 deficient mice were subjected to the experimental metastasis model. Lack of this endothelial glycocalyx proteoglycan disturbs the structure of the glycocalyx by reducing the amount of heparan sulphate moieties. As proteoglycans bidirectionally influence their signaling pathways, it might be expected that the reduced content of heparan sulphate moieties is accompanied by a reduction in hyaluronan content. As shown in Figure 2, when injected intravenously with B16F10 melanoma cells these syndecan-1 deficient mice showed a 3-fold reduced number of pulmonary tumor foci compared to wild type mice. These data show that genetic disruption of heparan sulphate moieties of the glycocalyx is anti-metastatic as well.

As already indicated, the glycocalyx is considered as an integrated and balanced carbohydrate layer in which both hyaluronan and heparan sulfate chains are key structural components. Importantly, our data show that targeting either hyaluronan (enzymatically by hyaluronidase treatment) or the heparan sulphate chains (genetic ablation of syndecan-1) of the glycocalyx leads to reduced experimental metastasis. As these two different interventions have a similar effect on experimental metastasis, our data imply that barrier protective-properties of the glycocalyx are less essential for metastasis than its functions in cancer cell adhesion or growth factor storage [28]. Future experiments are needed however to fully appreciate the role of specific components of the glycocalyx on metastasis and to elucidate the underlying mechanisms.

As mentioned before, some LMWHs protect against cancer progression in experimental animal models and clinical trails, including the B16F10 melanoma model of experimental metastasis. As suggested previously, these LMWHs may inhibit metastasis through competitive binding of heparanase or hyaluronidase thereby protecting the vascular endothelium and its barrier function from disruption caused by these enzymes. To assess whether the inhibitory effect of the administration of a LMWH on cancer progression are dependent on its protective effects on the glycocalyx, we compared the effect of enoxaparin administration on experimental metastasis in syndecan-1 deficient and wild type mice. As shown in Figure 2, enoxaparin injected intravenously at 30 min prior, and 6 and 12 h after cancer cell inoculation decreased the number of pulmonary tumor foci in wild type mice almost completely. Interestingly, LMWH administration also effectively reduced pulmonary tumor foci formation in syndecan-1 deficient animals (p = 0.02). These data show that the effect of this particular LMWH on secondary tumor formation is syndecan-1 independent and suggest that the cancer inhibiting effect of LMWHs may not be mediated by restoration of glycocalyx barrier function.

Some aspects of the experimental set-up require further comments. First, a lower amount of cancer cells was injected in the second experiment (Figure 2) in order to achieve lower numbers of pulmonary tumor foci that could be assessed more easily. Consequently, the wild type mice had less pulmonary tumor foci than in Figure 1. Moreover, syndecan-1-deficient mice appeared to be even better protected to secondary tumor formation than mice which received a single dose of hyaluronidase (approximately 80% versus 33% reduction in tumor foci in syndecan-1 deficient and hyaluronidase treated mice, respectively). This may imply that long term irreversible glycocalyx damage is more protective than temporally enzyme-mediated glycocalyx damage.

In conclusion, our data show that targeted interference of either hyaluronan or heparan sulfate limits experimental metastasis suggesting that the net effect of the glycocalyx is pro-metastatic. Moreover, the effect of enoxaparin on cancer progression and cancer cell metastasis is glycocalyx independent.

## Materials and Methods

### Hyaluronidase and heparin

Bovine testicular hyaluronidase (type IV-S; Sigma-Aldrich, St. Louis, MO) dissolved in 0.9% NaCl was administered intravenously in a dose of 100 units per mouse 1h prior to cancer cell inoculation [4]. LMWH (15 mg/kg; enoxaparin, Sanofi-Aventis, Paris, France) was injected 30 min prior to and 6, 12 and 24 h after cancer cell inoculation.

### Cells and cell culture

Murine B16F10 melanoma cells were obtained from the American Type Culture Collection (ATCC; Manassas, VA). Cells were cultured in Dulbecco Modified Eagle Medium (DMEM; Lonza, Verviers, Belgium) supplemented with 10% fetal calf serum (Sigma-Aldrich), 1% penicillin-streptomycin solution and 1% L-glutamine at 37°C as described before [29], [30]. Single cell suspensions were prepared from 2 mM EDTA-treated monolayer's which were washed and diluted in phosphate-buffered saline (PBS) prior to counting and inoculation. Cells were stored on ice until administration.

### Animals

Eight to ten week-old C57Bl/6 male mice (Charles River, Maastricht, The Netherlands) were maintained at the animal care facility of the Academic Medical Centre, Amsterdam, The Netherlands according to institutional guidelines. Syndecan-1 −/− male mice on a C57Bl/6 background were housed and bred in the Radboud University Nijmegen Medical Centre and handled as described [31]–[34]. Animal procedures were carried out in compliance with Institutional Standards for Humane Care and Use of Laboratory Animals. The institutional Animal Care and Use Committee approved all experiments (protocol number DIX101092).

### Experimental pulmonary metastasis model

Cancer cells (suspended in 200 µl PBS) were injected into the lateral tail vein as described before [35]–[37]. In the first experiment, 3.5×10^5^ cancer cells were administered per mouse, whereas in the second experiment 2.0×10^5^ cells were used in order to lower the amount of secondary tumor foci for purpose of countability. After 14 days, mice were sacrificed and lungs were prepared as described before [30]. Secondary tumor formation on the surface of the lungs was counted macroscopically in a blinded fashion with respect to the intervention. Experiments were performed with 8 mice per group.

### Statistical analysis

Statistical analysis was carried out in GraphPad Prism version 4.03. Data are expressed as means +/− SEM or medians with interquartile range. For normally distributed data, significance was assessed with the Student t-test. For not normally distributed data, non-parametric testing was performed using the Mann-Whitney test. Statistical significance was assumed when the p-value was <0.05.
